# Elevated 4R tau contributes to endolysosomal dysfunction and neurodegeneration in VCP-related frontotemporal dementia

**DOI:** 10.1093/brain/awad370

**Published:** 2023-10-26

**Authors:** Christy Hung, Rickie Patani

**Affiliations:** Human Stem Cells and Neurodegeneration Laboratory, The Francis Crick Institute, London NW1 1AT, UK; UCL Great Ormond Street Institute of Child Health, Zayed Centre for Research into Rare Disease in Children, London WC1N 1DZ, UK; Human Stem Cells and Neurodegeneration Laboratory, The Francis Crick Institute, London NW1 1AT, UK; Department of Neuromuscular Diseases, Queen Square Institute of Neurology, University College London, London WC1N 3BG, UK

**Keywords:** iPSC, SFPQ, FUS, tauopathy, lysosome

## Abstract

Frontotemporal dementia (FTD) and amyotrophic lateral sclerosis (ALS) are two incurable neurodegenerative diseases that exist on a clinical, genetic and pathological spectrum. The *VCP* gene is highly relevant, being directly implicated in both FTD and ALS. Here, we investigate the effects of *VCP* mutations on the cellular homoeostasis of human induced pluripotent stem cell-derived cortical neurons, focusing on endolysosomal biology and tau pathology. We found that *VCP* mutations cause abnormal accumulation of enlarged endolysosomes accompanied by impaired interaction between two nuclear RNA binding proteins: fused in sarcoma (FUS) and splicing factor, proline- and glutamine-rich (SFPQ) in human cortical neurons. The spatial dissociation of intranuclear FUS and SFPQ correlates with alternative splicing of the *MAPT* pre-mRNA and increased tau phosphorylation. Importantly, we show that inducing 4R tau expression using antisense oligonucleotide technology is sufficient to drive neurodegeneration in control human neurons, which phenocopies *VCP*-mutant neurons.

In summary, our findings demonstrate that tau hyperphosphorylation, endolysosomal dysfunction, lysosomal membrane rupture, endoplasmic reticulum stress and apoptosis are driven by a pathogenic increase in 4R tau.

## Teaser

Hung and Patani use a patient-derived stem cell model to uncover disease mechanisms in a type of dementia closely related to motor neuron disease called FTD. Specifically, they show that the increase of a particular type of tau protein—called the 4R isoform—drives degeneration of nerve cells, thereby causing the disease.

## Introduction

Amyotrophic lateral sclerosis (ALS) and frontotemporal dementia (FTD) are often referred to as ALS/FTD spectrum disorder.^1^ In FTD, fused in sarcoma (FUS), transactive response (TAR) DNA-binding protein 43 (TDP-43) and tau are pathological hallmarks.^2^ Valosin-containing protein (VCP/p97) is a ubiquitously expressed AAA+ ATPase with crucial roles in a multitude of intracellular functions^3^ and is directly implicated in both FTD and ALS. The first mutations in *VCP* were reported in 2004 to cause inclusion body myopathy (IBM), Paget’s disease (PDB) or FTD (collectively IBMPFD).^4^*VCP* mutations were subsequently reported in ALS and FTD.^5^ Indeed, a cohort of 231 patients carrying 15 distinct *VCP* mutations found that FTD was present in 30% of cases, whilst ALS (9%), Parkinson’s disease (4%) and Alzheimer’s disease (2%) were less represented.^6^ Over 30 mutations in *VCP* have been reported in ALS/FTD, particularly being associated with the FTD-TDP subtype.^7^*VCP* mutations can lead to a dominant negative loss-of-function or altered VCP function, possibly through the differential effects of different mutations on its oligomerization propensity or co-factor binding.^4,8^ IBMPFD mutations, A232E (located in the D1 ATPase domain) and R155H (located in the N-domain) cause increased ATPase activity compared to wild-type VCP.^9^ Conversely, the vacuolar tauopathy-causing mutation D395G, lies within the D1 ATPase domain of VCP and reduces ATPase activity by ∼30%, modestly impairing its disaggregase capability.^10^

While the predominant aggregate pathology in the VCP-multisystem proteinopathy (MSP) brain is reported to be TDP-43, several studies support the identification of tau-positive aggregates in the brain.^11,12^ The *VCP* R191Q mutation is associated with the presence of phosphorylated tau in specific brain regions such as the orbital cortex, caudate nucleus, amygdala, hippocampus, entorhinal cortex, locus coeruleus and raphe nuclei.^12^ Additionally, a previous study demonstrated that muscle biopsies from patients with *VCP* mutations revealed sarcoplasmic accumulation of phosphorylated-tau.^11^ Furthermore, several reports suggest that tau and TDP-43 pathology can co-exist in the context of neurodegeneration.^13–18^ The precise driving mechanism of MSP is not completely understood and the concept of ‘pathologic synergy’, where more than one protein contributes to pathogenesis, may be of relevance.^19^ However, there are also reports demonstrating minimal tau pathology in VCP-MSP cases, suggesting that tau may not be required for MSP pathogenesis. VCP has diverse molecular functions, reflected to some degree by the heterogeneity of clinical presentation caused by pathogenic variants in VCP. Further studies using post-mortem human brain samples from *VCP* mutation-carrying patients will be crucial to elucidate the relationship between *VCP* mutations, MSP and tau pathology, although these resources are exceptionally rare due to the low frequency of *VCP* mutations.

We recently showed that inhibiting the D2 ATPase of VCP using small molecular pharmacology reduces nuclear-to-cytoplasmic mislocalization of TDP-43, FUS and a range of other proteins in *VCP* mutant human induced pluripotent stem cell (hiPSC)-derived spinal motor neurons.^20,21^ Additionally, we and others observed the displacement of these canonical RNA-binding proteins (RBPs) from the nucleus more widely in familial and sporadic ALS.^22–24^ However, the underlying molecular mechanisms by which *VCP* mutations impair RBP interactions and splicing function, and their consequent effects on tau pathology, remain poorly understood in the context of progressive neurodegeneration, particularly in FTD. To address these issues, we investigated the consequences of *VCP* mutations on the cellular homeostasis of hiPSC-derived cortical excitatory neurons, focusing in particular on RBP localization and interaction, endolysosomal biology and tau pathology. The goal of our study was to determine the cell autonomous phenotypes in human cortical neurons carrying *VCP* mutations and identify the underlying mechanism driving neurodegeneration in this context.

## Materials and methods

### Experimental model and subject details

Control hiPSC lines: non-demented control (NDC),^25^ SFC840 (StemBANCC) and AD3.1 (StemBANCC). All *VCP* mutant lines: VCP mutant cell line 1 (R155C), VCP mutant cell line 2 (R155C) and VCP mutant cell line 3 (R191Q) were previously reported and characterized.^20,26^ This research was carried out in accordance with the UK Code of Practice for the Use of Human Stem Cell Lines.

### Directed differentiation to human cortical neuron culture

Directed differentiation of hiPSCs to the human excitatory cortical neurons was carried out as previously described.^27^ Briefly, dissociated hiPSCs were plated on six-well plates coated with GelTrex (Life Technologies) and neural induction was initiated by changing into culture medium that supports neuronal differentiation and neurogenesis, a 1:1 mixture of N2- and B27-containing media (N2B27) (supplemented with 1 μM dorsomorphin and 10 μM SB431542 to inhibit TGFβ signalling during neural induction). Media was replaced every 24 h. At Day 12, neuroepithelial cells were harvested with dispase and replated in laminin-coated plates with FGF2-containing media for 4 days. FGF2 was then withdrawn and dissociated using Accutase and neural progenitor cells were plated on GelTrex-coated plates. Plated neurons were maintained for up to 120 days with a medium change every 2–3 days.

### Protein extraction and western blot analysis

For immunoblotting, whole cell lysate protein was extracted with RIPA buffer (Sigma) supplemented with protease inhibitors (Sigma) and Halt phosphatase inhibitors (ThermoFisher Scientific). Protein quantification was performed using Precision Red Advanced Protein Assay buffer (Cytoskeleton, Inc.). Equal amounts of protein samples were then loaded onto a gel and separated on a 4–12% SDS-PAGE and transferred to PVDF membranes. Samples were then blocked with Licor blocking buffer at room temperature for 1 h, followed by primary antibody incubation overnight at 4°C. For detection, membranes were incubated with species-specific near infra-red fluorescent antibodies (IRDye, LI-COR) at room temperature for 1 h and imaged using a LI-COR Odyssey system. Antibodies used in this study are shown in Supplementary Table 1.

### Image acquisition and analysis

For immunostaining, cells were fixed in 4% paraformaldehyde (PFA) in PBS followed by permeabilization with Triton X-100 (Sigma). Fixed cells were blocked with 10% normal goat serum (Sigma) in PBS, probed with primary antibodies diluted in blocking solution and detected with goat anti-mouse, anti-chicken or anti-rabbit secondary antibodies coupled to Alexa Fluor 488, 594 or 647.

For the analysis of vesicle size, fixed cells were imaged on an iSIM microscope using a 150× oil objective with deconvolution. EEA1+, LAMP1+ and Galectin 1+ puncta quantifications were done using the spot detection function in the Imaris software. Surface masks were created using the TUJ11 or MAP2 staining to indicate neurons.

### Proximity ligation assay

Proximity ligation assay (PLA) was performed using NaveniFlex MR assay reagents according to the supplier’s guidelines (Navinci Diagnostics AB). Briefly, fixed cells were blocked and probed with primary antibodies diluted in blocking buffer overnight at 4°C followed by incubation with PLA probes and rolling circle amplification in the presence of ATTO 488 fluorophore. After multiple washing steps, cells were mounted with a VECTASHIELD antifade mounting medium with DAPI.

Cells were imaged with an instant structured illumination microscopy (iSIM) microscope using a 150× oil objective with deconvolution. Specific individual protein-protein interactions can be seen as green puncta. PLA+ puncta quantifications were done using the spot detection function in the Imaris software. Surface masks were created using MAP2 staining to indicate neurons.

### Antisense oligonucleotides

Splice-switching antisense oligonucleotides (ASOs) were designed with a phosphorothioate backbone and had the following modifications on a phosphorothioate (PS) backbone: uniformly modified with 2′-O-methoxyethyl nucleotides to improve nuclease resistance and prevent RNaseH-mediated degradation of *MAPT* mRNA. The ASOs were delivered to hiPSC-derived neurons by addition to extracellular media for a 10-day period. The 3R to 4R *MAPT* ASO sequence was GGCGCATGGGACGTGTGA.^28^

### Quantification and statistical analysis

Unless otherwise specified, statistics analysis was performed using GraphPad Prism (Version 9). Student’s *t*-test was used to compare differences between the two groups. Significance threshold was defined as adjusted *P*-value < 0.05 (*P < 0.05, **P < 0.01, ***P < 0.001, ****P < 0.0001). Error bars in all figures represent standard error of the mean (SEM). Unless otherwise stated, data shown are calculated from biological replicates (*n* = 3 control lines and *n* = 3 *VCP* mutant lines).

## Results

### Abnormal accumulation of enlarged endosomes and lysosomes in *VCP* mutant human cortical neurons

To study the effects of *VCP* mutations on neuronal homeostasis, we generated highly enriched and previously functionally validated human cortical excitatory neurons^27^ from hiPSCs derived from three independent cell lines from healthy individuals (termed ‘control neurons’ hereafter) and three independent cell lines with autosomal-dominant mutations in *VCP* that are causal for FTD (termed ‘*VCP* mutant neurons’ hereafter) (see Supplementary Fig. 1A and B for details of the cell lines). Both control and *VCP* mutant hiPSC-derived neurons expressed high levels of neuronal markers (TUJ1 and MAP2), confirming robust differentiation and no gross effect of the *VCP* mutation on cortical neuronal specification (Supplementary Fig. 1C and D). Previous studies have revealed endoplasmic reticulum (ER) stress and apoptosis in *VCP* mutant spinal cord motor neurons,^26^ which motivated us to address if similar pathology exists in *VCP* mutant cortical neurons. Indeed, we detected significantly increased expression of phosphorylated eIF2alpha (indicative of ER stress) and cleaved caspase 3 (signifying apoptosis) in *VCP* mutant cortical neurons compared with their control counterparts (Supplementary Fig. 1E–H).

**Figure 1:**
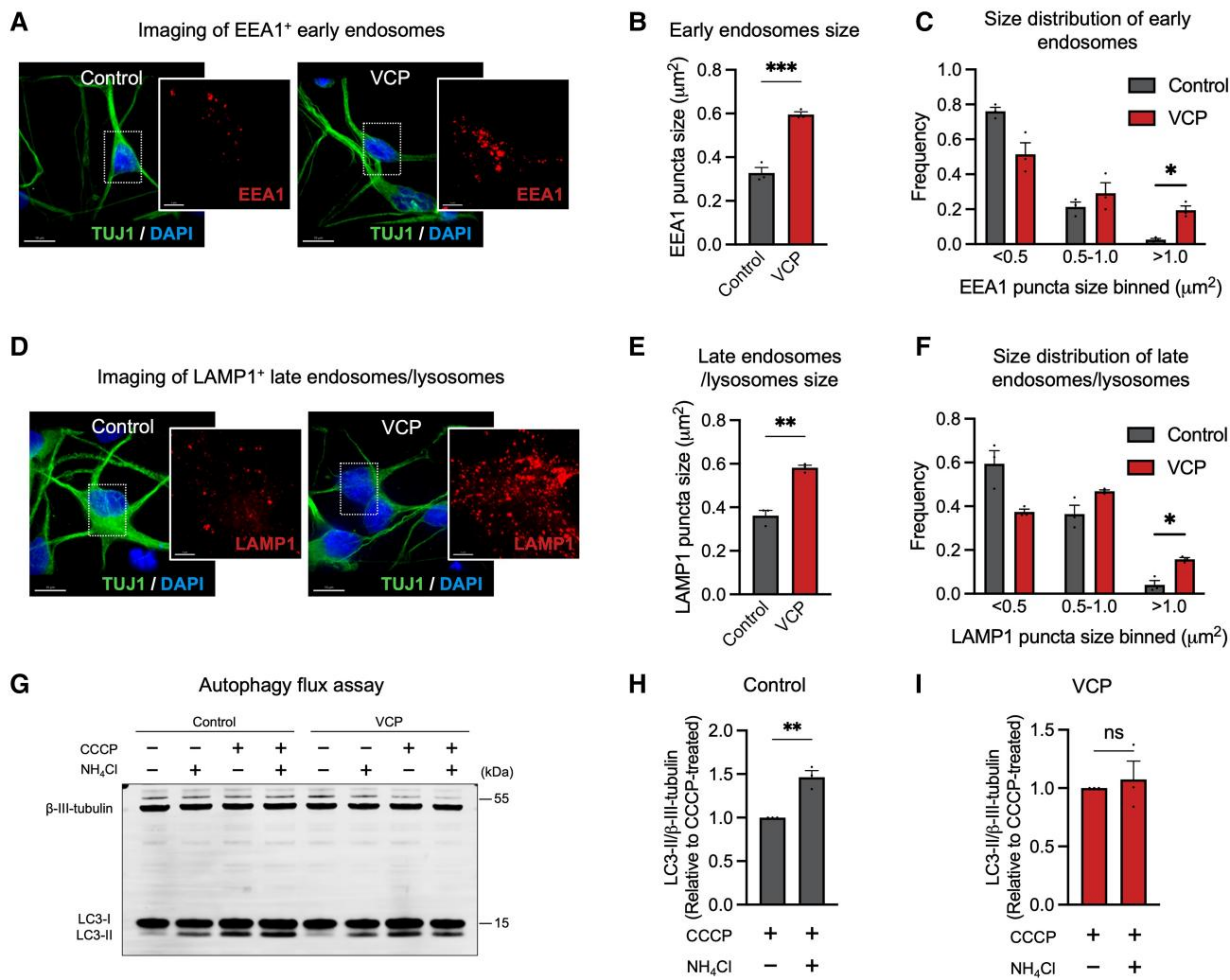
**Abnormal accumulation of enlarged endosomes and lysosomes in *VCP* mutant cortical neurons.** (**A**–**C**) Representative immunocytochemistry of human induced pluripotent stem cell (hiPSC)-derived neurons expressing EEA1 proteins (**A**) (red = EEA1; green = TUJ1; blue = DAPI). Scale bars = 10 μm. A significant increase in the average size of EEA1+ puncta (**B**) and frequency (**C**) of early endosomes with size > 1 μm^2^ in *VCP* mutant neurons (*n* = 3 control and *n =* 3 *VCP* mutant lines). (**D**–**F**) Representative immunocytochemistry of hiPSC-derived neurons expressing LAMP1 proteins (**D**) (red = LAMP1; green = TUJ1; blue = DAPI). Scale bars = 10 μm. A significant increase in the average size of LAMP1+ puncta (**E**) and frequency (**F**) of late endosomes/lysosomes with size > 1 μm^2^ in *VCP* mutant neurons (*n* = 3 control and *n =* 3 *VCP* mutant lines). (**G**) Autophagic degradation was calculated by comparing LC3-II levels following treatment with autophagy activators (CCCP) in the presence or absence of NH_4_Cl. Representative western blots of LC3I/II and neuron-specific β3-tubulin are shown (**G**). (**H** and **I**) Autophagosome degradation was significantly impaired in *VCP* mutant neurons, as calculated from the western blot analysis. A further elevation of LC3-II levels was only observed in control neurons (**H**) but not in *VCP* mutant neurons (**I**) (one-sample *t*-test) (*n* = 3 control and *n* = 3 *VCP* mutant lines).

Dysfunction of the endolysosome pathway is emerging as an important pathogenic process in FTD and ALS.^29^ VCP has previously been found to localize to the early endosome membrane, where it binds early endosomal autoantigen 1 (EEA1) to regulate the size of early endosomes.^30^ To investigate how *VCP* mutations impact the endolysosomal pathway, we used high resolution iSIM to measure the size of endogenous EEA1 (a well established early endosome protein^31^) (Fig. 1A). We observed a significant increase in the average size of early endosomes (EEA1+ puncta) in *VCP* mutant neurons compared with control neurons (Fig. 1B). In addition, we observed a significant increase in the frequency of larger early endosomes (>1 μm^2^) in *VCP* mutant neurons (Fig. 1C).

To further investigate whether *VCP* mutations impair the downstream events in the endocytic pathway, we measured the size and number of late endosomes/lysosomes by immunolabelling for the endogenous lysosomal-associated membrane protein 1 (LAMP1), which is one of the most abundant lysosomal components.^32^ We found a significant increase in the number of LAMP1+ puncta in *VCP* mutant neurons compared with their control counterparts (Fig. 1D and E). Distribution analysis of sizes revealed a significant increase in the frequency of larger puncta (>1 μm^2^) in *VCP* mutant neurons (Fig. 1F).

Given the dynamic nature of the lysosomal and autophagy pathway, we also measured autophagic flux using a previously reported quantitative approach.^33^ The basal LC3-II levels were similar between control and *VCP* mutant neurons (Fig. 1G). Autophagy was induced by mitochondrial respiratory chain uncoupler (CCCP) treatment, as demonstrated by an upregulation of LC3-II in the presence of both CCCP and NH_4_Cl compared to neurons treated with NH_4_Cl alone (Fig. 1G). However, we observed a further elevation of LC3-II levels only in control neurons (Fig. 1H) but not in *VCP* mutant neurons (Fig. 1I). Together, our data suggest that *VCP* R155C and R191Q mutations cause abnormal accumulation of enlarged endosomes and lysosomes accompanied by impaired autophagosome degradation.

### FUS and SFPQ exhibit both nuclear loss and intranuclear spatial dissociation in *VCP* mutant human cortical neurons

**Figure 2:**
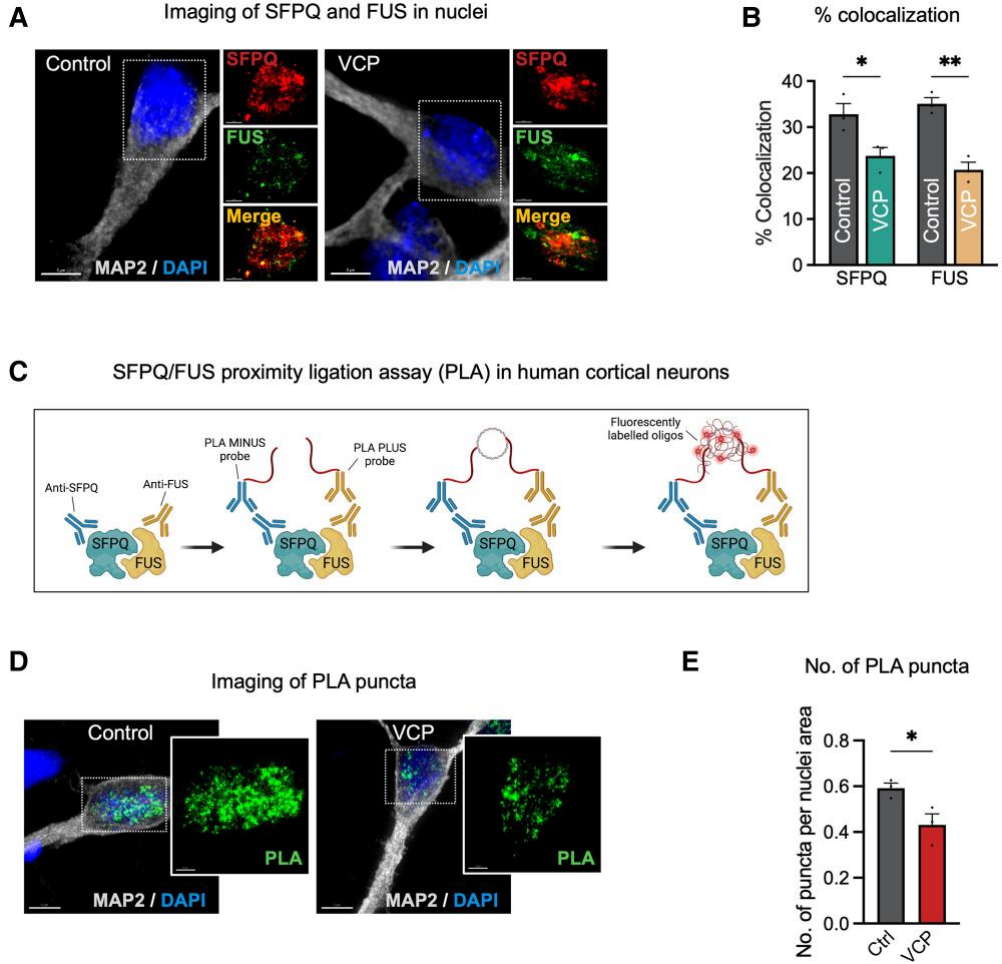
**FUS and SFPQ spatial dissociation in *VCP* mutant human cortical neurons.** (**A**) Representative immunocytochemistry of human induced pluripotent stem cell (hiPSC)-derived neurons expressing splicing factor, proline- and glutamine-rich (SFPQ) and fused in sarcoma (FUS) proteins (red = SFPQ; green = FUS; grey = MAP2; blue = DAPI). Scale bars = 5 μm. (**B**) A signification reduction in the % co-localization of SFPQ with FUS and FUS with SFPQ (*n* = 3 control and *n* = 3 *VCP* mutant lines). (**C**) Schematic of the proximity ligation assay (PLA) used in this study. (**D**) Representative immunocytochemistry of hiPSC-derived neurons after PLA reactions (green = PLA reactions; grey = MAP2; blue = DAPI). Scale bars = 5 μm. (**E**) A significant reduction in the number of PLA puncta per nuclei area in *VCP* mutant neurons compared with controls (*n* = 3 control and *n* = 3 *VCP* mutant lines).

FUS and SFPQ nuclear-to-cytoplasmic mislocalization has been observed in hiPSC spinal cord motor neurons carrying *VCP* mutations, along with the more established mislocalization of TDP-43.^20,21,26,34^ We examined the nuclear loss of FUS, SFPQ and TDP-43 in our *VCP* mutant cortical neuronal model of FTLD using high resolution iSIM combined with 3D deconvolution. We observed a significant nuclear loss of SFPQ, FUS and TDP-43 in *VCP* mutant compared to control cortical neurons (Supplementary Fig. 2). Previous studies have shown that FUS and SFPQ co-localize to form high molecular weight complexes in the nucleus that regulate alternative splicing of *MAPT* pre-mRNA, which encodes the protein tau.^35,36^ To investigate the effect of *VCP* mutations on FUS and SFPQ intranuclear co-localization, we quantified the localization of FUS and SFPQ using high resolution iSIM (Fig. 2A). We observed a significant reduction in co-localization of FUS and SFPQ in human cortical neurons carrying *VCP* mutations (Fig. 2B). To orthogonally validate this finding, we applied a well characterized PLA to study the co-localization between FUS and SFPQ (Fig. 2C). In this assay, PLA oligonucleotides + and − are directly conjugated to primary antibodies for FUS and SFPQ, respectively. This is followed by DNA hybridization and amplification steps. Interactions between FUS and SFPQ are then detected through the hybridization of fluorescently-labelled complementary oligonucleotides (Fig. 2D). We observed a significant decrease in the number of PLA puncta in *VCP* mutant neurons compared with their control counterparts (Fig. 2E), confirming aberrant interaction between FUS and SFPQ.

**Figure 3:**
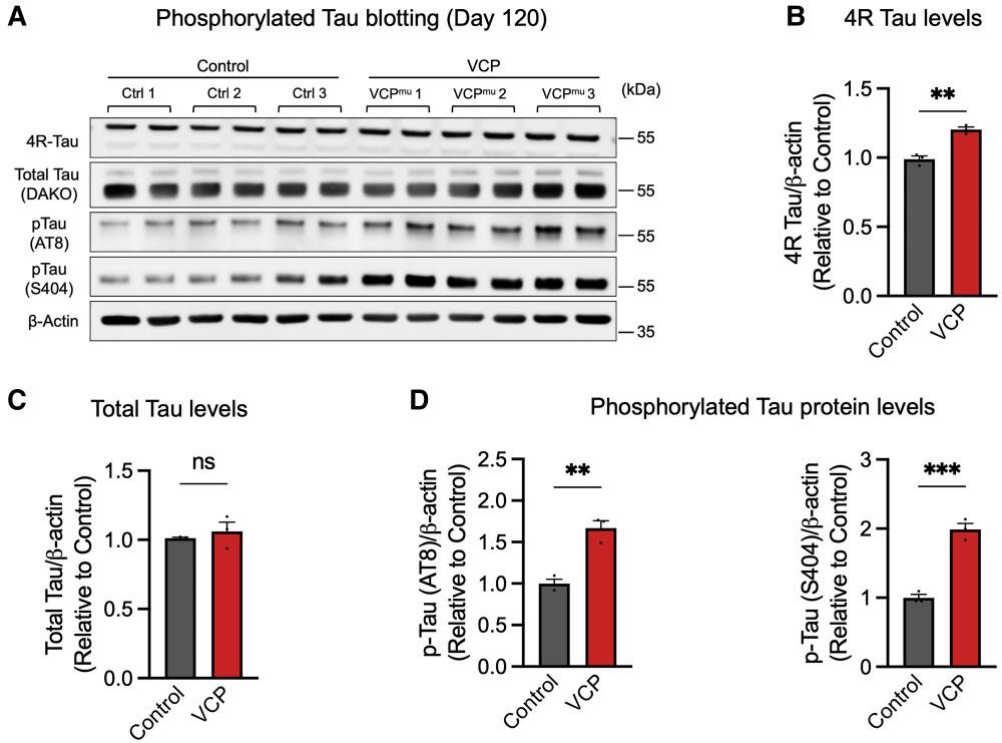
**Abnormal tau hyperphosphorylation in *VCP* mutant human cortical neurons.** (**A** and **B**) 4R tau levels were significantly higher in *VCP* mutant human cortical neurons. Representative western blots of total and phosphorylated tau are shown (**A**). Levels of 4R tau proteins were calculated relative to β-actin (**B**) (*n* = 3 control and *n* = 3 *VCP* mutant lines). (**C** and **D**) Total tau levels were unchanged (**C**). However, phosphorylated tau protein levels (**D**) were significantly higher in neurons generated from *VCP* mutant human induced pluripotent stem cells (hiPSCs) than those detected in three independent controls (*n* = 3 control and *n* = 3 *VCP* mutant lines).

Consistent with previous findings that FUS/SFPQ spatial dissociation leads to deregulated alternative splicing of *MAPT* pre-mRNA,^35^ we observed a marked increase in 4R tau levels in neurons carrying *VCP* mutations with no changes in total tau levels (Fig. 3A–C and Supplementary Fig. 3). Tau phosphorylation was also increased in *VCP* mutant neurons compared with controls, including at Ser404 and Ser202/Thr205 (AT8) (Fig. 3D), epitopes frequently associated with tau-mediated dementias.^37^

### 3R to 4R MAPT splice-switching antisense oligonucleotides recapitulate molecular phenotypes of VCP-related FTD

**Figure 4:**
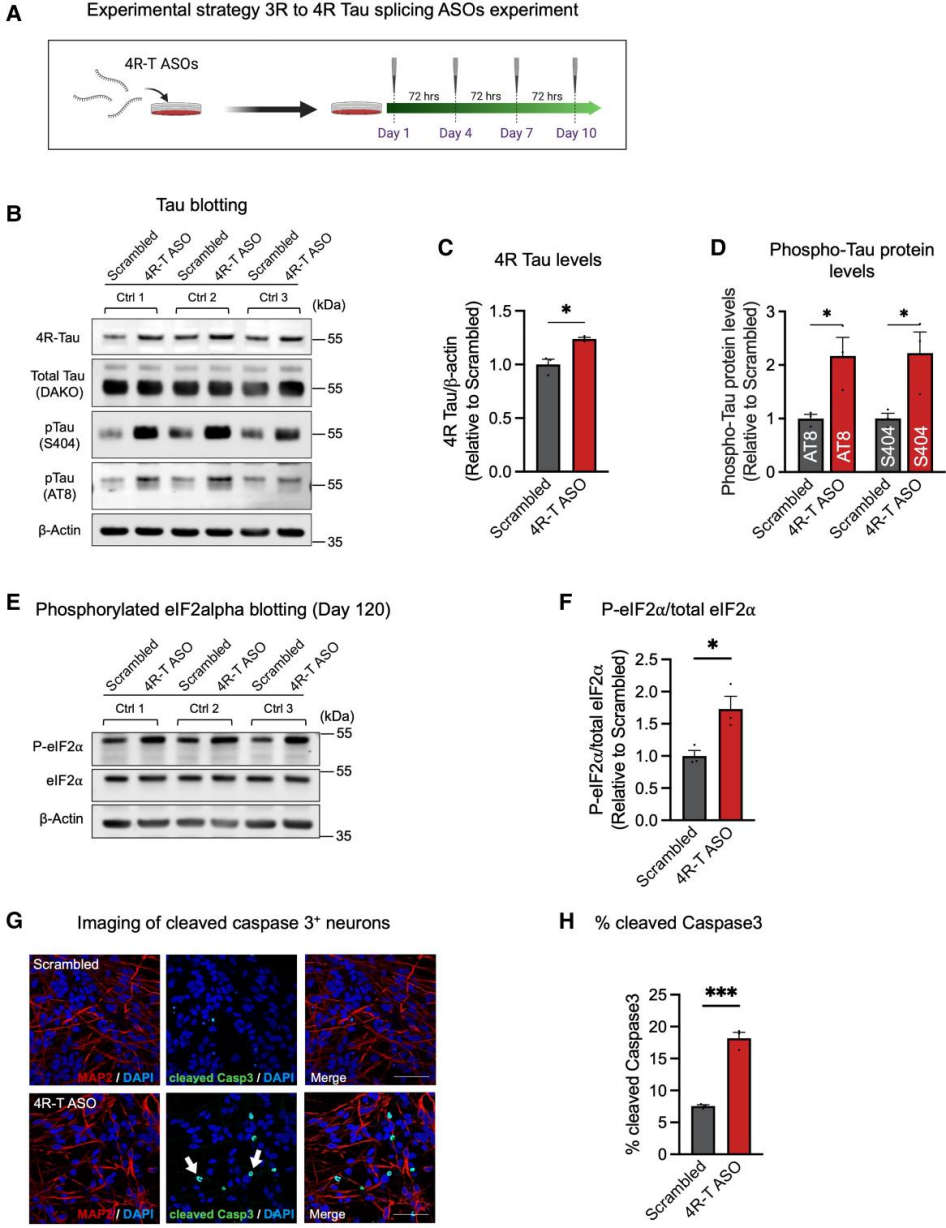
**3R to 4R *MAPT* ASOs recapitulate tau hyperphosphorylation, ER stress and apoptosis.** (**A**) Experimental strategy 3R to 4R tau splice-switching antisense oligonucleotides (ASOs) experiment. (**B**–**D**) Representative western blots of 4R tau, total tau and phosphorylated tau are shown (**B**). 4R tau levels were significantly higher in neurons treated with 4R-T ASOs (**C**). This was accompanied by a significant increase in phosphorylated tau protein levels (**D**) (*n* = 3 control lines). (**E** and **F**) Phosphorylated eIF2alpha levels were significantly higher in human cortical neurons treated with 4R-T ASOs. Representative western blots of phospho-eIF2alpha and total eIF2alpha are shown (**E**). Ratios of phospho-eIF2alpha to total eIF2alpha were calculated relative to scrambled controls (**F**) (*n* = 3 control lines). (**G** and **H**) Representative immunocytochemistry of human induced pluripotent stem cell (hiPSC)-derived neurons expressing cleaved caspase 3 (white arrows) (**G**) (red = MAP2; green = cleaved caspase 3; blue = DAPI). Scale bars = 50 μm. A significant increase in the percentage of cleaved caspase 3+ cells in neurons treated with 4R-T ASOs (**H**) (*n* = 3 control lines).

The data reported above demonstrate that *VCP* mutations are associated with spatial dissociation of intranuclear SFPQ and FUS, which deregulates alternative splicing of *MAPT* pre-mRNA in human cortical neurons. To formally test whether an increase in 4R tau is sufficient to drive endolysosome defects and abnormal tau phosphorylation, we applied tau isoform switching ASOs to increase neuronal 4R tau protein levels in human cortical neurons (Fig. 4A).

To do so, we used a 3R to 4R *MAPT* mRNA splice-switching ASO designed with a phosphorothioate backbone and uniformly modified with 2′-*O*-methoxyethyl nucleotides that has been previously shown to effectively target the human *MAPT* mRNA in human tau-expressing mice^28^ and hiPSC-derived astrocytes^38^ to increase the 4R:3R tau ratio. ASOs (3.5 μM) were delivered to control neurons by gymnosis for a 10-day period, which resulted in a significant increase in 4R tau protein levels (Fig. 4B and C and Supplementary Fig. 4). We observed a significant increase in tau phosphorylation in 3R to 4R splicing ASO-treated neurons compared with scrambled controls, including at Ser404 and Ser202/Thr205 (AT8) (Fig. 4D). Our data indicate that an increase in 4R tau drives abnormal tau phosphorylation.

To test whether an increase in 4R tau protein also phenocopies the increased expression of phosphorylated eIF2alpha (p-eIF2alpha) and cleaved caspase 3 observed in *VCP* mutant cortical neurons (Supplementary Fig. 1E–H), we assessed the levels of total eIF2alpha and phosphorylated eIF2alpha by western blotting (Fig. 4E). We detected significantly increased expression of p-eIF2alpha in neurons treated with 3R to 4R splicing ASOs compared with scrambled controls (Fig. 4E and F). In addition, we also found a significantly higher proportion of cells positive for cleaved caspase 3 in 3R to 4R splicing ASO-treated neurons (Fig. 4G and H). Together, our data suggest that an increase in 4R tau is sufficient to recapitulate molecular phenotypes of *VCP*-related FTD, including abnormal tau phosphorylation, elevated phosphorylation of eIF2alpha and apoptosis.

### Elevated 4R tau drives endolysosomal dysfunction and lysosomal membrane rupture

**Figure 5:**
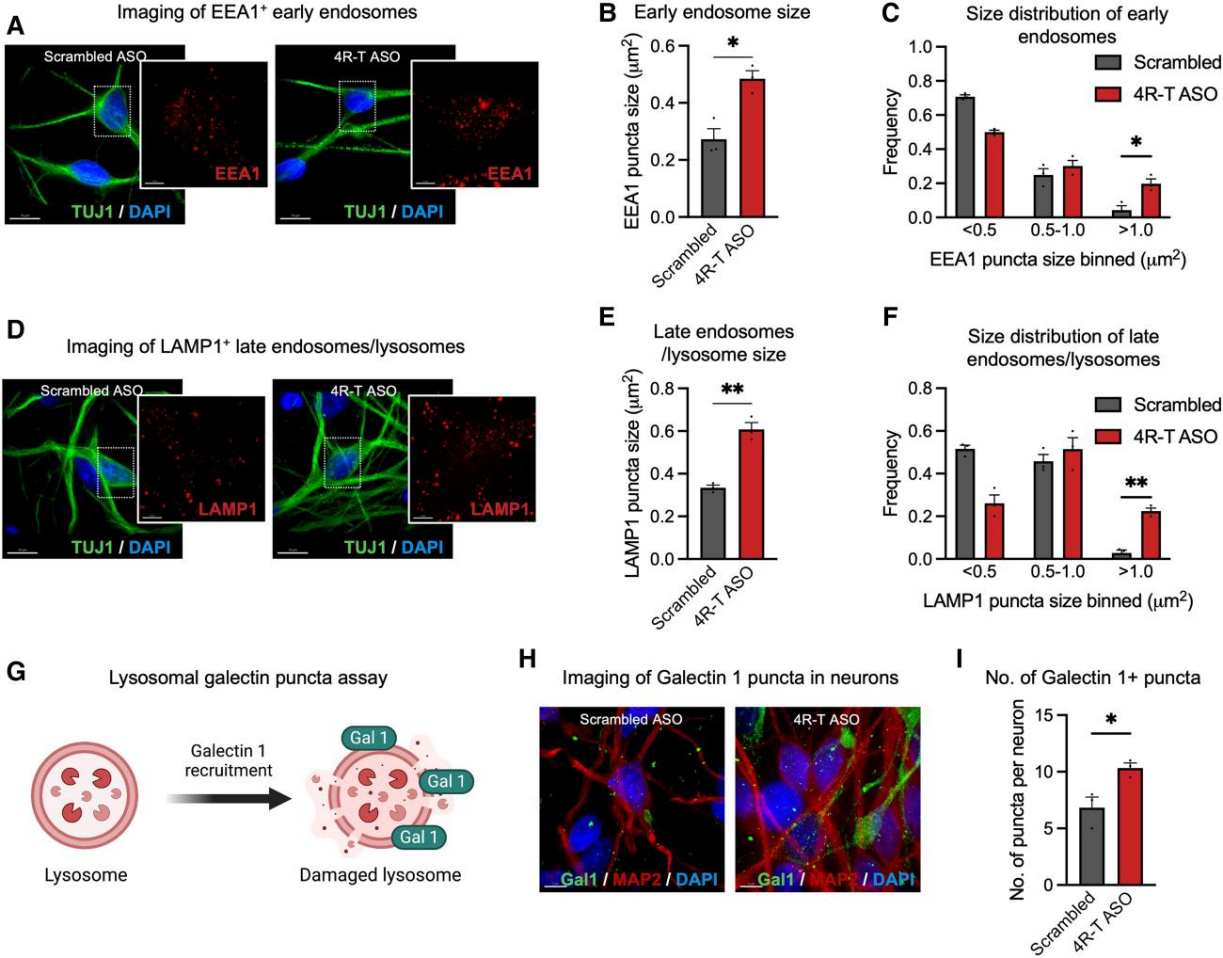
**3R to 4R *MAPT* ASOs result in endolysosomal dysfunction.** (**A**–**C**) Representative immunocytochemistry of human induced pluripotent stem cell (hiPSC)-derived neurons expressing EEA1 proteins (**A**) (red = EEA1; green = TUJ1; blue = DAPI). Scale bars = 10 μm. A significant increase in the average size of EEA1+ puncta (**B**) and frequency (**C**) of early endosomes with size > 1 μm^2^ in neurons treated with 4R-T antisense oligonucleotides (ASOs) (*n* = 3 control lines). (**D**–**F**) Representative immunocytochemistry of hiPSC-derived neurons expressing LAMP1 proteins (**D**) (red = LAMP1; green = TUJ1; blue = DAPI). Scale bars = 10 μm. A significant increase in the average size of LAMP1+ puncta (**E**) and frequency (**F**) of late endosomes/lysosomes with size > 1 μm^2^ in neurons treated with 4R-T ASOs (*n* = 3 control lines). (**G**) Schematic of the lysosomal galectin puncta assay used in this study. (**H**) Representative immunocytochemistry of hiPSC-derived neurons expressing Galectin 1 proteins (green = Galectin 1; red = MAP2; blue = DAPI). Scale bars = 5μm. (**I**) A significant increase in the number of Galectin 1+ puncta in neurons treated with 4R-T ASOs compared with scrambled controls (*n* = 3 control lines).

To test whether an increase in 4R tau is sufficient to drive endolysosome defects, we used high resolution iSIM as described above to measure the size of endogenous EEA1 and LAMP1. We observed a significant increase in the average size of early endosomes (EEA1+ puncta) (Fig. 5A and B) and frequency of larger early endosomes (>1 μm^2^) (Fig. 5C) in human neurons treated with 3R to 4R splicing ASOs.

We also found a significant increase in the number of LAMP1+ puncta (Fig. 5D and E), accompanied by a significant increase in the frequency of larger puncta (>1 μm^2^) (Fig. 5F) in human neurons treated with 3R to 4R splicing ASOs compared with scrambled controls.

The lysosomal membrane is normally protected by highly glycosylated membrane proteins, such as LAMP1.^39^ We hypothesized that the increase in lysosomal size may make the lysosomes more prone to rupture, resulting in the release of hydrolytic enzymes from ruptured lysosomes into the cytosol. To test this, we measured lysosomal membrane permeabilization using a well established and highly sensitive lysosomal galectin puncta assay^40^ (Fig. 5G). To detect lysosomal damage, we used high resolution iSIM to quantify the number of Galectin 1 puncta (Fig. 5H). Galectin 1 belongs to the family of glycan-binding proteins and gets recruited to the sites of endolysosomal leakage.^40^ We found that 3R to 4R splice-switching ASOs resulted in a significant increase in the number of Galectin 1+ puncta (Fig. 5I). Together, our data show that 4R tau dominance results in endolysosomal dysfunction and lysosomal membrane rupture in human cortical neurons.

## Discussion

FTD and ALS are two devastating and untreatable neurodegenerative diseases that exist on a spectrum of shared clinical, pathological and/or genetic features.^1^ In particular, the *VCP* gene is highly relevant, with mutations being associated with both FTD and ALS. FUS, SFPQ and tau pathology are well recognized hallmarks of ALS-FTD spectrum disorders. However, the underlying molecular mechanisms of how *VCP* perturbs FUS and SFPQ interaction and subcellular localization, and consequent effects on tau pathology remain poorly understood.

In this study, we systematically investigated the effects of FTD-causing *VCP* mutations on neuronal homeostasis using a highly enriched and functionally validated hiPSC-derived, patient-specific cortical neuron model. We first demonstrated that familial *VCP* mutations cause abnormal accumulation of enlarged endosomes and lysosomes and aberrant interaction between FUS and SFPQ in human cortical neurons. The spatial dissociation of intranuclear FUS and SFPQ correlates with dysregulation of alternative splicing of *MAPT* pre-mRNA and increased tau phosphorylation in human *VCP* mutant cortical neurons. Importantly, we show that increased 4R tau using splice-switching antisense oligonucleotide technology is sufficient to drive neurodegeneration in human cortical neurons, including increased tau phosphorylation, endolysosomal dysfunction, lysosomal membrane rupture, endoplasmic reticulum stress and apoptosis.

Mislocalization of RBPs represents a hallmark of FTLD spectrum disorders, raising the hypothesis that a loss of nuclear function as a possible underlying disease mechanism. Indeed, SFPQ-knockdown mice exhibit FTLD-like phenotypes^35^ and motor neurons lacking SFPQ degenerate by exhibiting a ‘dying back’ phenotype reminiscent of ALS in zebrafish disease models.^41^ However, it is important to also consider intranuclear spatial dissociation of RBP complexes in the molecular pathogenesis of FTLD spectrum disorders. These two mechanisms are not mutually exclusive, as demonstrated in our study, and it is likely that the spatial dissociation of FUS and SFPQ is exacerbated by nuclear loss of these two RBPs.

Prior elegant work that motivated our study includes reports of SFPQ and FUS existing in a high molecular weight complex and regulating the splicing of *MAPT.*^35,36^ Crucially, this work showed the necessity of 4R tau for FTD phenotypes in mice in which knock down of FUS or SFPQ was performed.^35^ A subsequent study from the same group revealed the nuclear spatial dissociation of FUS and SFPQ in a range of disorders using post-mortem tissue in ALS/FTD (both FUS and TDP-43 pathological subtypes), progressive supranuclear palsy and corticobasal degeneration.^36^ Our study extends this work in several ways. First, we used a human stem cell model and showed that these phenotypes occur cell autonomously in cortical excitatory neurons. Second, we revealed additional phenotypes in these cells including endolysosomal dysfunction, aberrant autophagic flux, ER stress and cell death. Third, we showed that increased 4R tau is sufficient to drive this pathogenic cascade in control human excitatory cortical neurons, suggesting that it might be a viable therapeutic candidate for *VCP* mutation-related FTD.

Dysfunction of the endolysosome pathway is emerging as an important pathogenic process in FTD and ALS. A recent study reported that two FTD-ALS genes, *C9orf72* and *TBK1*, converge on the endolysosomal pathway to induce TDP-43 pathology and degeneration.^29^ While our study focuses on *VCP*, several other genes associated with FTD and ALS, such as *CHMP2B*,^42^*GRN*^43^ and *TMEM106B*,^44^ have been shown to affect the endolysosomal system. Crucially, our study raises the prospect of targeting 4R tau therapeutically to ameliorate the pathogenic cascade of events including increased tau phosphorylation, endolysosomal dysfunction, lysosomal membrane rupture, ER stress and apoptosis. Taken together, these data raise the possibility that endolysosomal dysfunction represents a convergent pathogenic ‘design principle’ shared by FTD and ALS.

## Supplementary Material

awad370_Supplementary_Data

## Data Availability

The data that support the findings of this study are available from the corresponding author, upon reasonable request.
