# Supplementary material for: Elevated 4R tau contributes to endolysosomal dysfunction and neurodegeneration in VCP-related frontotemporal dementia
Source: Brain. 2023 Oct 26;147(3):970–9. doi: 10.1093/brain/awad370 (PMC10907086; doi:10.1093/brain/awad370)
Supplement: awad370_Supplementary_Data [file awad370_supplementary_data.pdf]

## Supplementary information

| | |
|---|---|
| Mouse monoclonal anti-FUS | Santa Cruz Biotechnology |
| Rabbit monoclonal anti-SFPQ | abcam |
| Rabbit polyclonal anti-Tubulin β-3 | BioLegend |
| Mouse monoclonal anti-Tubulin β-3 | BioLegend |
| Mouse monoclonal anti-β-actin | Sigma |
| Rabbit polyclonal anti-LAMP1 | abcam |
| Rabbit polyclonal anti-LC3B | Sigma |
| Chicken polyclonal anti-MAP2 | abcam |
| Rabbit monoclonal anti-EEA1 | abcam |
| Rabbit polyclonal anti-Tau | DAKO |
| Rabbit polyclonal anti-4R tau | Cosmo Bio |
| Mouse monoclonal anti-3R tau | Sigma |
| Mouse monoclonal anti-phospho-tau pS202/T205 | Thermo Fisher Scientific |
| Rabbit monoclonal anti-phospho-tau S404 | abcam |
| Rabbit polyclonal anti-eIF2alpha | Cell Signaling |
| Rabbit polyclonal anti-phospho-eIF2alpha | Cell Signaling |
| Rabbit polyclonal anti-Galectin 1 | abcam |

**Supplementary table 1. Details of antibodies used in this study.**

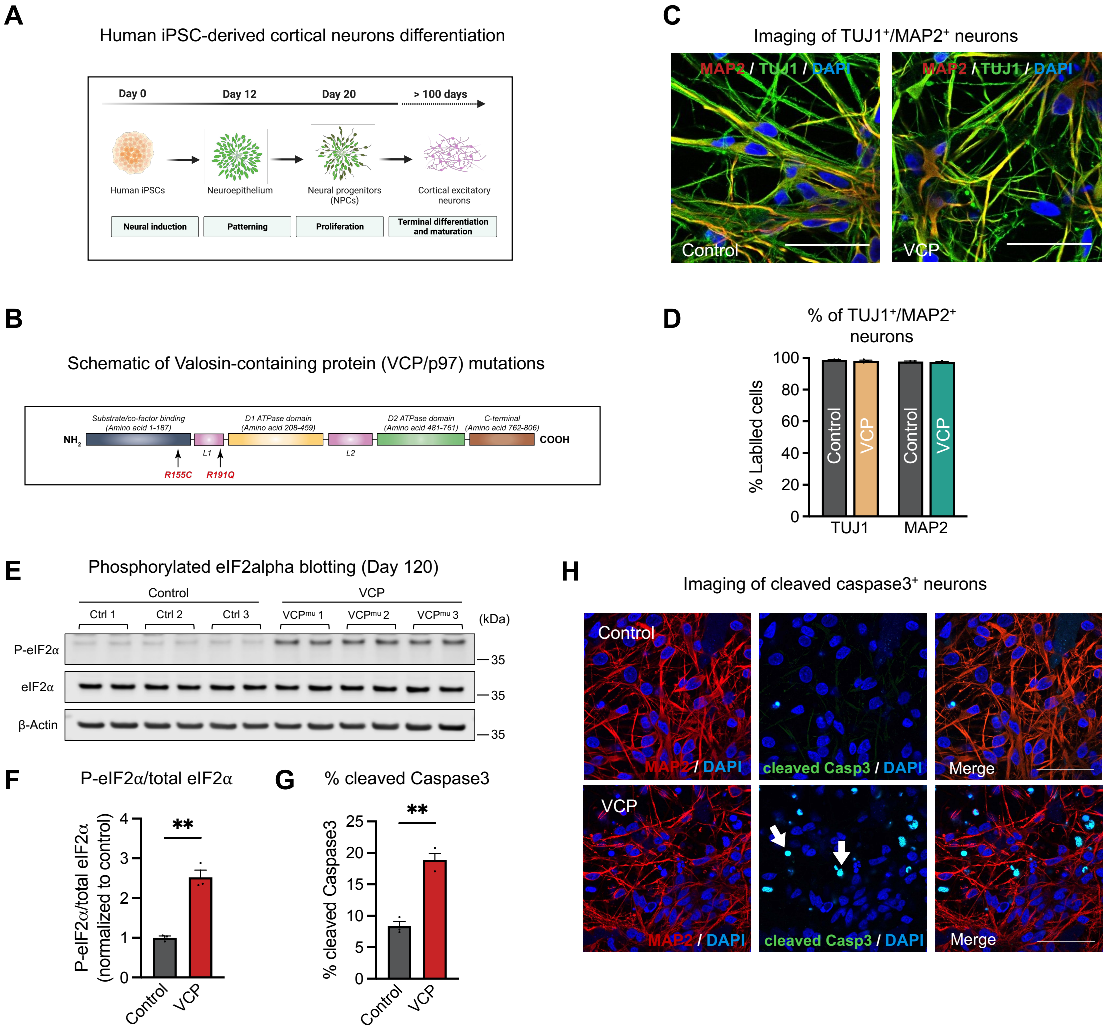


**Figure S1. Increased apoptosis and ER stress in *VCP* mutant cortical neurons**

(A) Schematic of hiPSC-derived cortical neurons differentiation.

(B) Schematic of the VCP mutations.

(C) Representative immunocytochemistry of neurons from control or carrying a *VCP* mutation (green, β3-tubulin; red, MAP2 and blue, DAPI). Scale bars, 50 μm.

(D) Analysis of TUJ1+ and MAP2+ neurons in control and *VCP* mutant neurons. (n = 3 control and 3 *VCP* mutant lines).

(E and F) Phosphorylate eIF2alpha levels were significantly higher in *VCP* mutant human cortical neurons. Representative western blots of phospho-eIF2alpha and total eIF2alpha are shown (E). Ratio of phospho-eIF2alpha to total eIF2alpha were calculated relative to control (F). (n = 3 control and 3 *VCP* mutant lines).

(G and H) A significant increase in the percentage of cleaved caspase 3+ cells in *VCP* mutant neurons compared to controls (G). Representative immunocytochemistry of hiPSC-derived neurons expressing cleaved caspase 3 (white arrows) (H) (red, MAP2; green, cleaved caspase 3; blue, DAPI). Scale bars, 50 μm. (n = 3 control and 3 *VCP* mutant lines).

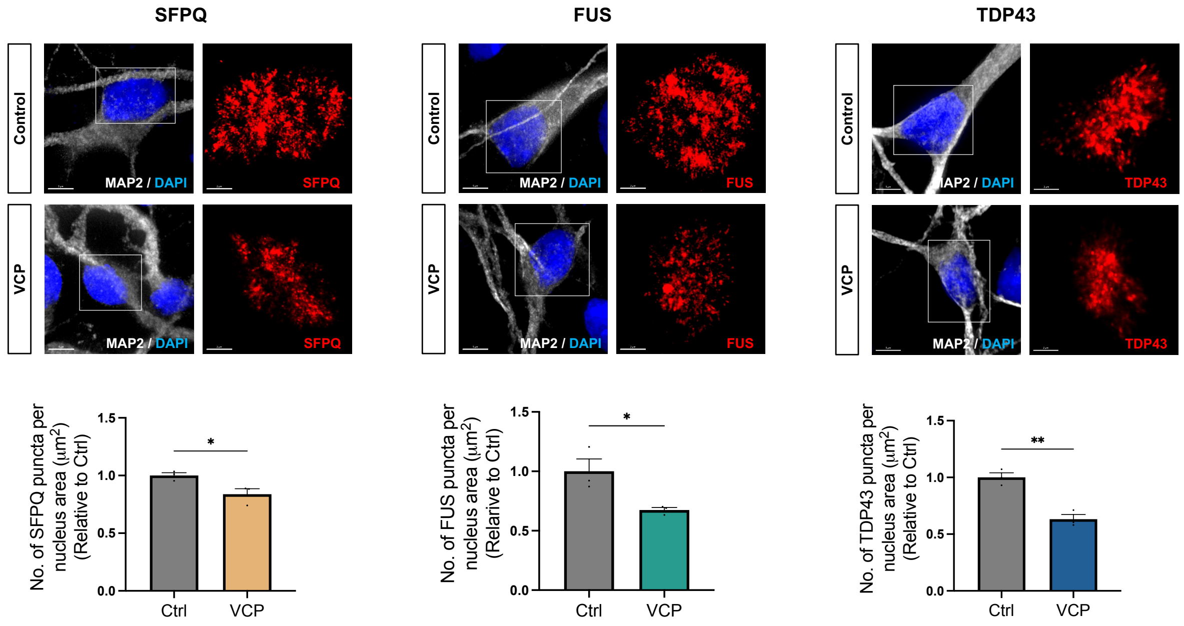


**Figure S2. Nuclear loss of FUS, SFPQ and TDP-43 in *VCP* mutant cortical neuronal model of FTLD.** Representative immunocytochemistry of hiPSC-derived cortical neurons expressing SFPQ (left), FUS (middle) and TDP43 (right) proteins (scale bars, 5 µm). A signification reduction in the no. of SFPQ (left), FUS (middle) and TDP43 (right) puncta per nuclear area. (n = 3 control and 3 *VCP* mutant lines).

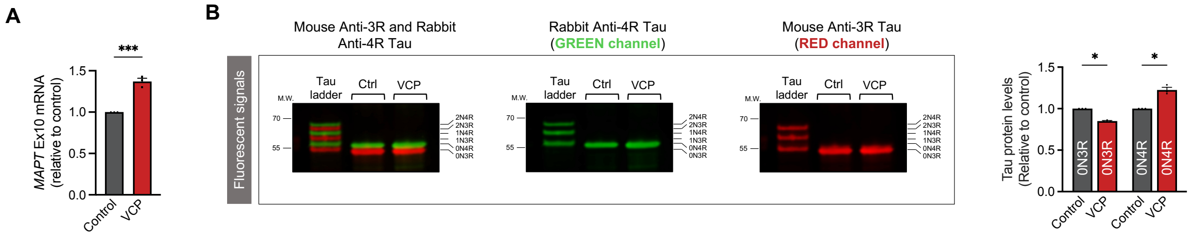


**Figure S3. Increase in 4R tau levels in neurons carrying *VCP* mutations.**
(A) RT-qPCR showing the relative abundance of *MAPT* mRNA containing the exon 10 (encoding for the additional microtubule-binding repeat).
(B) Tau isoforms with three (3R) or four (4R) microtubule-binding regions were detected by western blot analysis of dephosphorylated protein extracts from hiPSC-derived control and *VCP* mutant cortical neurons.

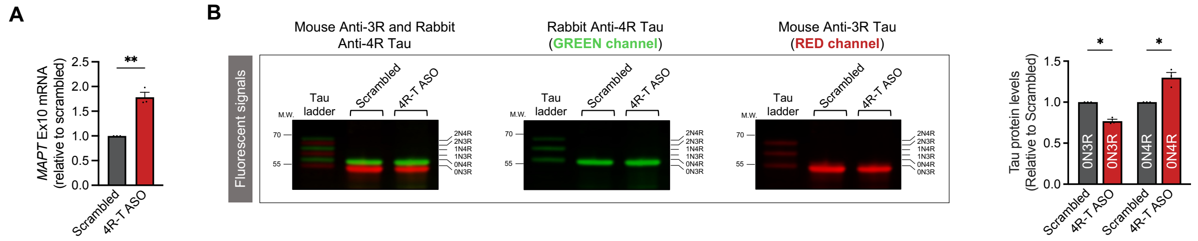


**Figure S4. Increase in 4R tau levels in neurons treated with 3R to 4R *MAPT* splice-switching ASOs.**

(A) RT-qPCR showing the relative abundance of *MAPT* mRNA containing the exon 10 (encoding for the additional microtubule-binding repeat).

(B) Tau isoforms with three (3R) or four (4R) microtubule-binding regions were detected by western blot analysis of dephosphorylated protein extracts from hiPSC-derived cortical neurons treated with scrambled ASOs or 4R-T ASOs.

Original western blot for **Figure 1H**

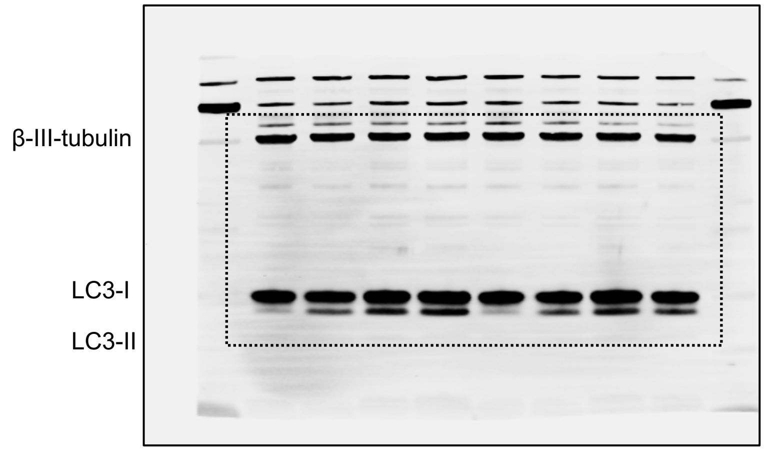


Original western blots for **Figure 3A**

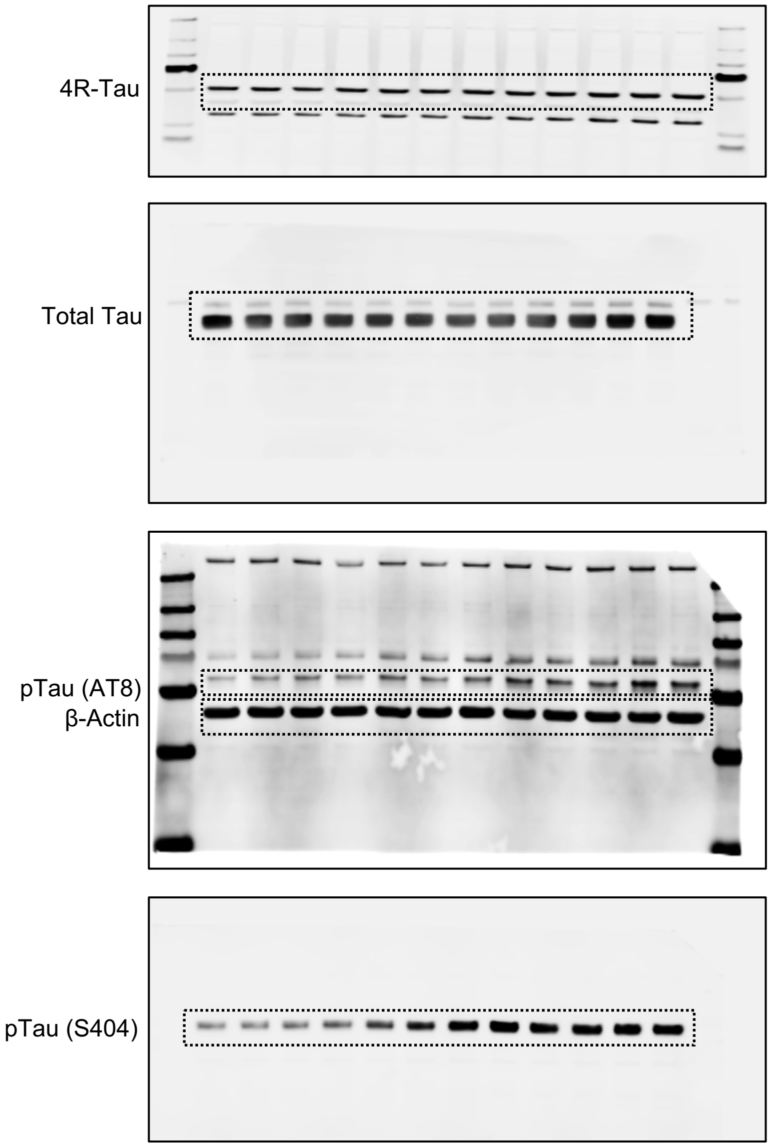

<u>Original western blots for **Figure 4B**</u>

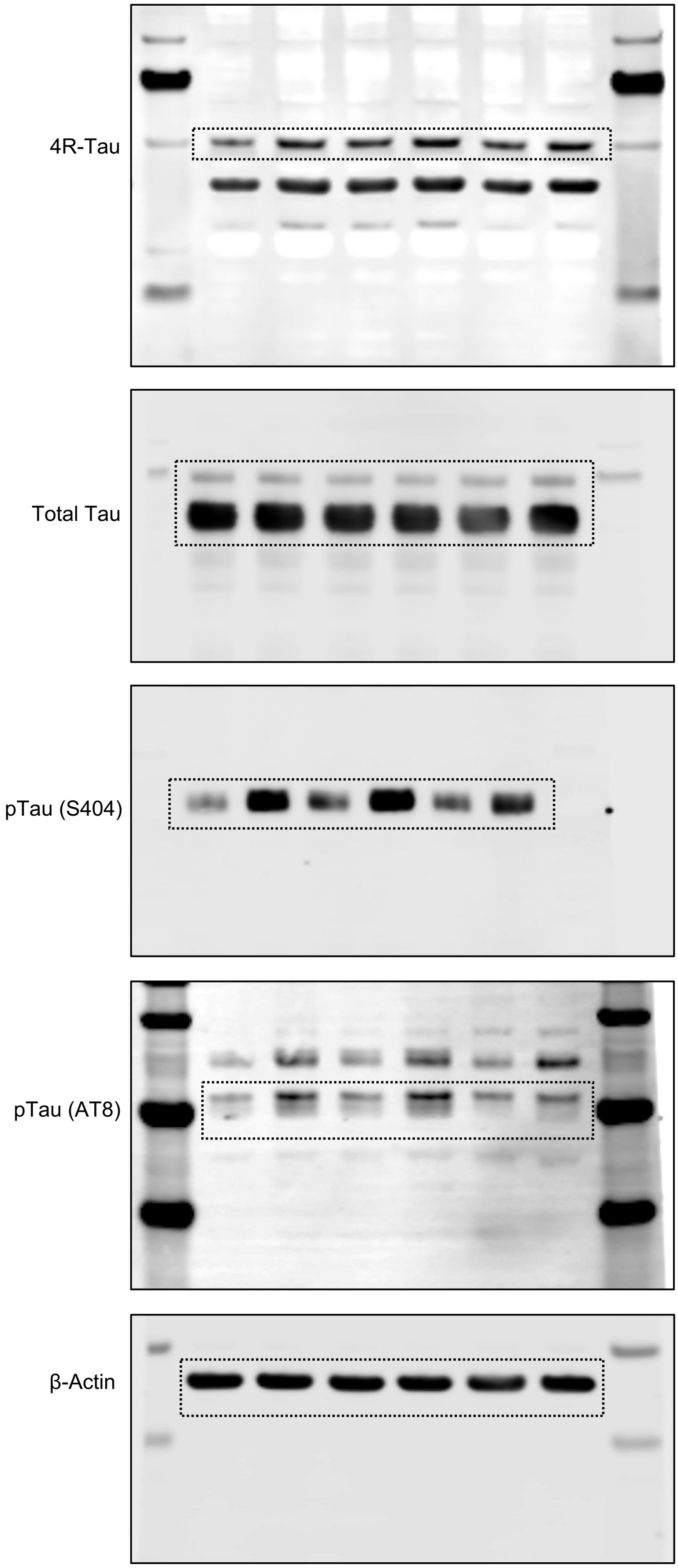

Original western blots for **Figure 4E**

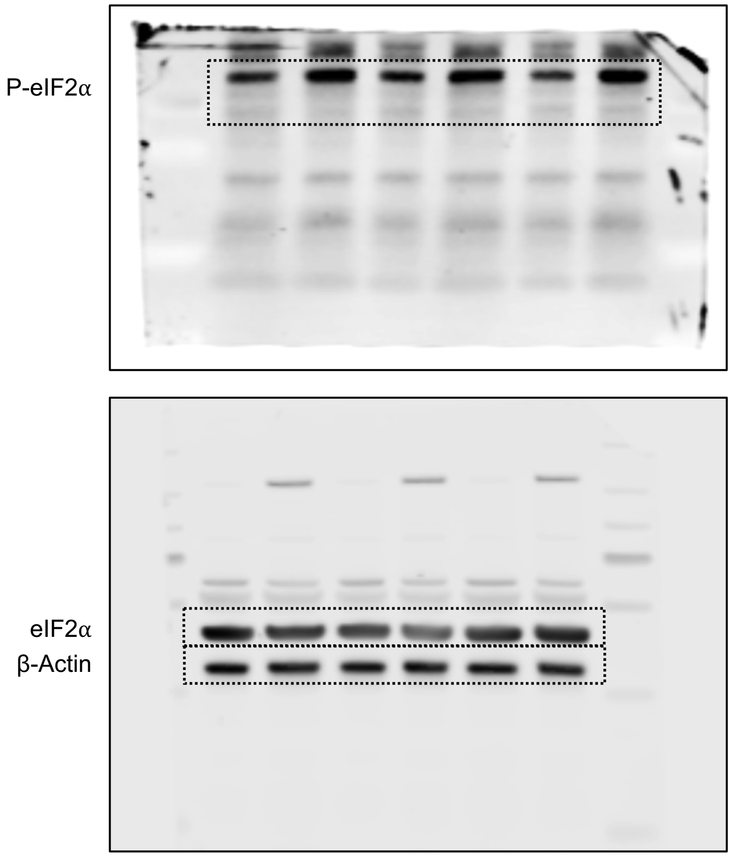


**Figure S5. Full western immunoblots.** Blots for each figure are indicated. Dashed lines indicate bands used as representative images.
